# Towards Personalized Medicine: Microdevice-Assisted Evaluation of Cancer Stem Cell Dynamics and Treatment Response

**DOI:** 10.3390/cancers17121922

**Published:** 2025-06-10

**Authors:** Eduardo Imanol Agüero, Silvia María Gómez López, Ana Belén Peñaherrera-Pazmiño, Matías Tellado, Maximiliano Sebastián Pérez, Betiana Lerner, Denise Belgorosky, Ana María Eiján

**Affiliations:** 1 Universidad de Buenos Aires, Facultad de Medicina, Instituto de Oncología Ángel H. Roffo, Área de Investigación, Buenos Aires C1417DTB, Argentina; imanol@stamm.bio (E.I.A.); sgomezlopez@institutoroffo.uba.ar (S.M.G.L.); 2 Consejo Nacional de Investigaciones Científicas y Técnicas (CONICET), Ciudad Autónoma de Buenos Aires C1425FQD, Argentina; 3 Agencia Nacional de Promoción de la Investigación, el Desarrollo Tecnológico y la Innovación, Fondo para la investigación Científica y Tecnológica (FONCyT), Ministerio de Innovación, Ciencia y Tecnología (MINCyT), Ciudad Autónoma de Buenos Aires C1425FQD, Argentina; 4 Centro de Investigación Biomédica (CENBIO), Facultad de Ciencias de la Salud Eugenio Espejo, Universidad UTE, Quito 170527, Ecuador; ana.penaherrera@ute.edu.ec; 5 VetOncologia-Clínica Oncológica Veterinaria, Buenos Aires C1408BGD, Argentina; vetoncologia@gmail.com; 6 Centro IREN, Universidad Tecnológica Nacional (UTN), Buenos Aires B1706EAH, Argentina; maxperez@fiu.edu (M.S.P.); belerner@fiu.edu (B.L.); 7 Collaborative Research Institute Intelligent Oncology (CRIION), 79117 Freiburg im Breisgau, Germany; 8 Department of Electrical and Computer Engineering, Florida International University, Miami, FL 33174, USA

**Keywords:** cancer stem cells, sphere formation assay, microfluidic device, chemotherapy response, personalized oncology, tumor-derived spheres, sustainable development goal 3

## Abstract

**Background/Objectives:** Cancer stem cells (CSCs) represent a minor yet critical subpopulation within tumors, endowed with self-renewal and differentiation capacities, and are implicated in tumor initiation, progression, metastasis, therapeutic resistance, and recurrence. Reliable in vitro functional assays to characterize CSCs are pivotal for the development of personalized oncology strategies. This study sought to establish and validate a microfluidic device (MD) platform for the enrichment, functional assessment, and therapeutic evaluation of CSC populations derived from experimental models and primary tumor samples. **Methods:** Murine (LM38LP) and human (BPR6) breast cancer cell lines were cultured within MDs to promote sphere formation. CSC enrichment was confirmed through the expression analysis of pluripotency-associated genes (Oct4, Sox2, Nanog, and CD44) by quantitative PCR (qPCR) and immunofluorescence. Sphere number, size, and gene expression profiles were quantitatively assessed before (control) and after chemotherapeutic exposure. To validate the MD platform against conventional scale, parallel experiments were performed in 12 well plates. To extend translational relevance, three primary canine tumor samples (solid thyroid carcinoma, simple tubular carcinoma, and reactive lymph node) were mechanically disaggregated and processed within MDs for CSC characterization. **Results:** The MD platform enabled the consistent enrichment of CSC populations, showing significant modulation of sphere growth parameters and stemness marker expression following chemotherapeutic treatment. Beyond its comparability with conventional culture, the MD also supported immunofluorescence staining and allowed real-time monitoring of individual cell growth. Sphere formation efficiency (SFE) and CSC marker expression were similarly demonstrated in primary veterinary tumor cultures, highlighting the device’s cross-species applicability. **Conclusions:** Microfluidic-based sphere assays represent a robust, reproducible, and scalable platform for the functional interrogation of CSC dynamics and therapeutic responses. This methodology holds great promise for advancing CSC-targeted therapies and supporting personalized oncology in both human and veterinary settings.

## Simple Summary

Cancer stem cells are a distinct subpopulation within tumors that possess the ability to self-renew, resist treatment, and drive tumor recurrence. Their identification and characterization are essential for developing more effective and personalized cancer therapies. In this study, we established a microdevice-based platform that enables the growth of three-dimensional cancer spheres from both established cancer cell lines and primary tumor samples. This system provides a controlled environment that requires minimal sample volumes and improves the evaluation of cancer stem cell behavior and drug response. We demonstrated its utility by assessing the effect of chemotherapy on cancer spheres and by analyzing tumors from veterinary patients. Our findings suggest that this platform could be a valuable tool for advancing research in human and veterinary oncology, supporting the development of targeted treatment strategies.

## 1. Introduction

Cancer is a complex and globally increasing disease. In 2020, there were close to 20 million new cases and 9.7 million cancer-related deaths worldwide based on GLOBOCAN estimates of incidence and mortality worldwide for 36 cancers in 185 countries [1]. Projections indicate that by 2040 the annual incidence will rise to 29.5 million new cases, with 16.4 million cancer-related deaths. Beyond its significant health burden, cancer also has profound impacts on patients’ quality of life, their families, and the global economy. Standard treatments include tumor surgery, chemotherapy, and radiotherapy [2]. However, despite advancements in diagnosis and therapy, many patients fail to respond effectively and experience local or distant recurrences [3,4]. Therefore, the development of novel tools to identify treatment-sensitive tumors is essential for advancing personalized medicine.

Among the factors contributing to tumor recurrence and treatment resistance, cancer stem cells (CSCs) have garnered significant attention. As quiescent and pluripotent cells, CSCs can remain dormant during treatment and, under specific conditions, regenerate tumors [5,6,7,8]. Although their prevalence varies by tumor type, CSCs represent a dynamic and minor population within the tumor microenvironment. They exhibit distinct characteristics, including the ability to grow under non-adherent conditions, form spheres, and express pluripotency markers such as Sox2, Nanog, Oct4, and CD133, among others [9,10,11,12]. Given these features, CSCs are an intriguing target for developing novel diagnosis and therapeutic approaches in oncology.

A major challenge in cancer research is the limited availability of tumor biopsy samples, which requires the development of microdevices (MDs) for prospective studies using minimal biological material. Several MD models designed to support CSC growth have been reported [13,14]. One such MD, previously described, features an inlet, an outlet, and 72 wells where CSCs can form spheres and respond to chemotherapy. This design allows for high-throughput analysis at a small volume. However, since it lacks multiple inlets, a separate MD is required for each experimental condition. Thus, designing new MDs tailored for specific biological and therapeutic studies is crucial.

This study presents a novel MD with six independent channels, enabling the simultaneous analysis of multiple cancer cell lines or primary tumor cultures. The device also facilitates the assessment of chemotherapeutic responses by evaluating sphere formation efficiency, growth rate, and pluripotency gene expression through qPCR and immunofluorescence. This work represents a foundational step toward developing a diagnostic tool for predicting CSC response to chemotherapy.

## 2. Materials and Methods

### 2.1. Design and Fabrication of MDs

The MD was made using polydimethylsiloxane (PDMS, Sylgard 184, Dow Corning, Midland, MI, USA) on a 1 mm-thick PDMS support, following previously described protocols [13,14]. The device consists of six channels, each containing five wells (1.5 mm in diameter), with an inlet and outlet (5 mm in diameter) created using a biopsy hole puncher. Devices were sterilized with ethylene oxide gas and degassed for 24 h before use. Cell suspensions were injected using a micropipette, and distribution was verified.

### 2.2. Cell Culture

The human breast cancer cell line BRP6 was derived from a Luminal A breast tumor sample obtained at the Instituto de Oncología “Ángel H. Roffo”. Murine LM38-LP [15] and BRP6 breast cancer cell lines, confirmed mycoplasma-free by PCR, were maintained as monolayers in complete medium (CM): DMEM-F12 (Gibco™ LS31800022, Waltham, MA, USA) supplemented with 2 mM L-glutamine, 1 mg/mL penicillin–streptomycin, and 10% fetal bovine serum (FBS). Cells were incubated at 37 °C with 5% CO_2_.

To evaluate inhibitory concentrations (IC_50_), cells were seeded in 96-well culture plates at a density of 2.5 × 10⁴ cells/well in CM and incubated overnight at 37 °C in a humidified atmosphere containing 5% CO_2_. The following day, the medium was replaced with fresh medium containing 2% FBS and increasing concentrations of the cytotoxic compounds. After 24 h of treatment, cell viability was assessed using the MTS/PMS assay (CellTiter 96^®^ AQueous MTS Reagent, Promega™, Promega Corporation2800, Woods Hollow Road, Madison, WI, USA), following the manufacturer’s instructions. Absorbance was measured at 490 nm using a BioTek^®^ Synergy™ LX multi-mode microplate reader. Dose–response curves were generated, and IC_50_ values were defined as the drug concentration that reduced absorbance to 50% of the untreated control. For the LM38-LP cell line, the IC_50_ values were 1.5 µM for doxorubicin (Doxo) and 0.5 µM for paclitaxel (Ptx). For the BRP6 cell line, IC_50_ values were 10 µM for Doxo and 12 µM for Ptx.

### 2.3. CSC-Sphere Formation Assay

For 3D culture, cells were grown in serum-free medium (SFM) supplemented with 2% B-27^®^ (Gibco™ 17504-044, USA), 20 ng/mL recombinant human fibroblast growth factor (rhFGF) (Gibco™ PHG00024, USA), and 20 ng/mL recombinant human epidermal growth factor (rhEGF) (Promega™ G502A, Madison, WI, USA). Spheres were cultivated in low-attachment conditions at high dilution. CSC sphere formation was analyzed in MDs by seeding 100 cells/µL SFM, followed by treatment with Doxo or Ptx for seven days. Culture medium was refreshed every three days by simple droplet diffusion. As a growth control, a parallel assay was conducted in a 12-well low-attachment culture plate (MW12) containing 3000 LM38-LP cells/mL in SFM. Sphere-forming efficiency (SFE) was calculated as the ratio of formed spheres to seeded cells. Sphere size was measured every two days based on diameter, as described by Belgorosky et al. [16].

### 2.4. Immunofluorescence Assay into MD

Immunofluorescence staining was performed to assess pluripotency marker expression in the MD, following established protocols [13]. Spheres were fixed in 4% formalin in 0.1 M PBS and incubated for one hour in a blocking solution (5% FBS in PBS). Primary antibodies for Oct-4 (1:50, ab18976, Abcam^®^, Cambridge, UK) or Alexa-Fluor^®^ 488 CD44 (103016, BioLegend^®^, San Diego, CA, USA) were incubated overnight at 4 °C. The secondary antibody (1:750, ab150077, Abcam^®^) was incubated within the MD for two hours at room temperature. Nuclei were stained with DAPI (1:10,000, sc-3598, Santa Cruz Technologies™, Dallas, TX, USA) for 20 min. Images were captured using a Nikon^®^ Eclipse E-600 fluorescence microscope (Nikon eclipse, Nikon corporation, Tokyo, Japan) with 20× Plan Fluor objectives and a Nikon^®^ Digital camera (Nikon H-III AP FDX-35).

### 2.5. Molecular Analysis by qPCR- RNA Purification from MD

To determine mRNA levels of Oct4, Sox2, Nanog, and CD44, spheres were eluted from the MD, and total RNA was extracted using the GenElute™ Universal Total RNA Purification Kit (Sigma-Aldrich^®^, St. Louis, MO, USA). Reverse transcription of 1 µg total RNA was performed using the SuperScript™ III Reverse Transcriptase kit (Invitrogen^®^, Carlsbad, CA, USA). qPCR was conducted using a CFX96™ Real-Time PCR Detection System (Bio-Rad Laboratories Inc., Hercules, CA, USA) with TransStart^®^ Green qPCR SuperMix (Trans^®^, China AQ101-01, Beijing, China), following the manufacturer’s instructions. Glyceraldehyde-3-phosphate dehydrogenase (GAPDH) was used as a housekeeping gene, and relative expression levels were calculated using the 2−ΔΔCt method. Primer sequences are listed in Table 1.

### 2.6. Tumorsphere Isolation from Tumor Samples

Canine and mouse tumors were cultured in MD. LM38-LP fat-pad tumors were grown in BALBc female mice aged 6–8 weeks (*n* = 8) by inoculating 2 × 10^5^ cells in 100 uL. The experimental unit was the individual animal. All mice were included if they successfully developed a palpable tumor following LM38-LP cell inoculation. Randomization was not used to allocate animals to different groups, as only one experimental group was included in this study. To minimize potential confounding variables, animals were housed under uniform conditions (same cage type, room, and light/dark cycle), and procedures were performed in the same order and by the same personnel whenever possible. No other confounders were identified or controlled. The investigators conducting the animal procedures and those assessing the outcomes were not blinded to the group allocation, as this study included a single experimental group. Tumor size was measured regularly using calipers. No procedures involving treatment or surgery were performed that required analgesia or anesthesia. No adverse events, either expected or unexpected, were observed during the course of the experiment. Humane endpoints were not established, as this study was designed to conclude before animals showed any signs of distress or illness. Mice were euthanized once tumors reached an appropriate size for sphere isolation.

Twenty-two days after inoculation, eight independent tumor samples were collected under aseptic conditions, in tubes containing cold DMEM-F12 with penicillin and streptomycin. Mechanical disaggregation using 70 μm nylon cell strainer (JetBiofil^®^ CSS013070, Guangzhou, China) in DMEM-F12 culture medium was performed. The obtained cell suspension was washed and suspended in 10 mL of DMEM-F12. Cells were counted and seeded into the MD as explained in 2.3 with or without IC50 Ptx, and sphere formation was assessed after 9 days.

In order to evaluate the versatility of the MD in culture of veterinary patients, three canine tumors were processed, in the same condition described above, seeding 3 × 10^5^ cells/mL, evaluating %SFE and expression of pluripotency genes.

### 2.7. Ethics Statement

The BRP6 breast cancer cell line was established from a human tumor sample, and the protocol approved by the Institutional Ethics Committee from Instituto de Oncología Angel H. Roffo (IOAHR) according to the Declaration of Helsinki, with the corresponding informed consents from each patient. Mice were obtained from the animal facility of the IOAHR and handled in accordance with the ARRIVE guidelines. The mice were housed in a controlled environment with a 12 h light/dark cycle, with ad libitum access to food and water. Animals were monitored daily to assess general health and wellbeing. The experimental procedures were planned in advance and conducted following institutional guidelines for animal research.

Protocols were approved by the Institutional Review Board of IOAHR CEI: 2020 and 02/03/2022 and CICUAL 01/2022.

### 2.8. Statistical Analysis

All experiments were performed in triplicate and were repeated at least three times independently. The results are expressed as a plot from GraphPad Prism^®^ 8.0.1 and are presented as mean values ± standard deviations. Statistical differences were determined using one-way and two-way analysis of variance (ANOVA), *p* < 0.05 was considered significant.

## 3. Results

### 3.1. MD Description

**Figure 1:**
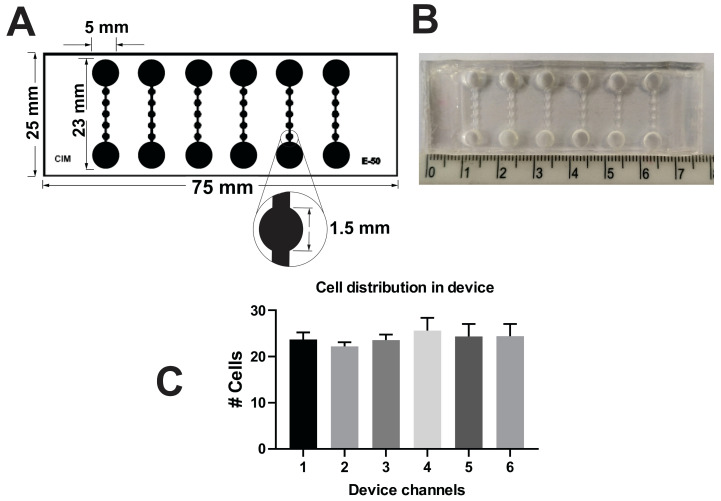
*Description of MDs.* (**A**) Schematic representation of the MD layout, featuring six independent linear channels. Each channel contains five circular wells (1.5 mm in diameter) connected between an inlet and an outlet reservoir (5 mm in diameter), allowing controlled media flow. (**B**) Photograph of the actual MD. (**C**) Distribution of LM38-LP. Cells counts were performed in six randomly selected wells across different channels to assess seeding uniformity.

The MD consisted of six independent channels containing inlet and outlet wells at the corners of each channel, with a diameter of 5 mm. Between these, there is a channel with five 1.5 mm diameter chambers, enabling anchorage-independent cell growth (Figure 1A,B). Having independent channels allows the possibility of analyzing different conditions within the MD. Nevertheless, in order to do so, we first performed a study of cell distribution within the channels. It was possible to demonstrate that cell density does not vary between channels (Figure 1C).

### 3.2. Sphere-Forming Efficiency and Diameter Under Chemotherapeutic Treatment

**Figure 2:**
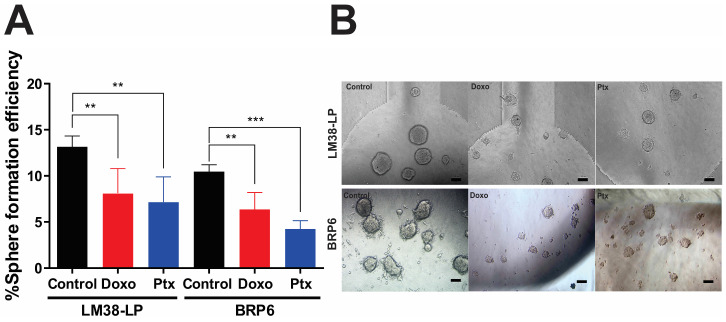
Chemotherapeutic effect in LM38-LP and BRP6 sphere proliferation. A total of 100 cells/μL from each cell line were seeded in the microfluidic device and received the chemotherapeutic treatment with doxorubicin or paclitaxel for 7 days. Analysis of cytotoxic effects in (**A**) percentage of sphere-forming efficiency (%SFE) and (**B**) representative phase-contrast images of each condition. One-way blocked ANOVA with Dunnett’s multiple comparisons” (** *p* < 0.05, and *** *p* < 0.0005). Scale bar 100 µm.

The sphere-forming assay (SFE) is a currently accepted technique to evaluate CSC. Here, two breast cancer cell lines, murine LM38-LP and human BRP6, were used to evaluate whether the MD is a suitable platform for CSC growth. Each cell line was seeded on an MD, using the first two channels for the control condition, the next two for the Doxo treatment, and the last ones for growth with Ptx. Figure 2 shows sphere growth in MD, and Table 2 summarizes the findings for both the number and size of spheres with Doxo or Ptx compared to the control. When comparing the growth of spheres, from LM38-LP, between MD and MW12, we observe that the efficiency is lower when growing on the devices (54% less). Furthermore, upon analyzing the size of the spheres growing on both surfaces, we notice that although fewer spheres grow on the MD, their sizes are 31% larger than on the MW12. This suggests that this platform may be doing a more “effective” selection of CSCs. On the other hand, the %SFE within the MD is similar for the human line BRP6 and the murine line LM38-LP. However, a lower response is seen in the human line for both chemotherapy drugs, possibly related to cell biology.

These results show the versatility of the MD for the analysis of CSC growth and their response to treatment in murine and human lines.

### 3.3. Expression of CSC-Associated Genes in LM38-LP and BRP6 Cell Lines

To assess the enrichment of pluripotency markers in the growing spheres, we extracted total RNA from spheres cultured in both MD and MW12. As shown in Table 3, when 26 LM38-LP spheres were cultured in MD, we obtained 17.4 μg of RNA, an amount adequate for molecular analyses by qPCR. Conversely, culturing cells in MW12 resulted in the generation of 28 spheres, yielding 81.4 μg of RNA. This indicates a substantial difference in the RNA obtained from spheres cultured on both platforms, despite starting with a similar number of spheres. The variance could potentially stem from incomplete elution of the MD spheres, leading to a lower final yield of total RNA. Nevertheless, despite the significantly lower RNA yield from MD, it maintains excellent quality and is sufficient for subsequent molecular analyses.

**Figure 3:**
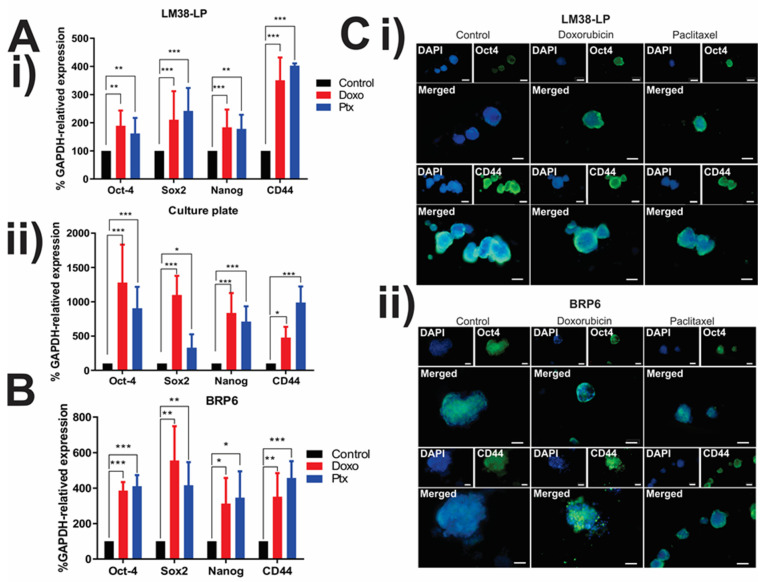
Expression of pluripotency genetic markers in spheres under chemotherapeutic treatment by qPCR and IF. (**A**) Percentage of GAPDH-related expression of pluripotency markers Oct-4, Sox2, Nanog, and CD44 mRNA by qPCR in LM38-LP spheres growing in (**i**) MDs and (**ii**) culture plate and (**B**) in BRP6 spheres. (**C**) Images of IF Alexa ^®^488 staining for Oct4 and CD44 in (**i**) LM38-LP and (**ii**) BRP6 spheres inside the device. Both techniques were used for control spheres and 7-day treated ones. Scale bar 100 μm (One-way ANOVA, *** *p* < 0.0005, ** *p* < 0.005, and * *p* < 0.05).

In Figure 3, the expression of Oct-4, Sox2, Nanog, and CD44 pluripotency genes in the spheres growing within MD (Figure 3A,B) was assessed. A significant upregulation in the expression of these markers was noted following treatment with Doxo or Ptx, in comparison to untreated spheres in both lines. Furthermore, an increase in pluripotency markers was also observed for the LM38 LP line growing in MW12.

MDs have proven to be a valuable platform for conducting immunofluorescence (IF) assays, offering a faster, simpler, and more efficient approach compared to MW12. By employing conventional immunofluorescence techniques with specific antibodies, it became feasible to analyze the expression of two pluripotency markers (Oct4 and CD44) in spheres from both lines, with or without treatment, without the need to isolate the spheres. The expression of both markers in tumor spheres was demonstrated, affirming that MDs serve as an apt platform for CSC enrichment and subsequent immunophenotyping (Figure 3A,B). MDs proved to be a useful platform for conducting immunofluorescence (IF) within them, in a quicker, simpler, and more efficient manner than in MW12.

The results from these assays suggest that treatment with these drugs may increase the pluripotent capacity of therapy-resistant clones. However, we cannot rule out the possibility that chemotherapy is selecting for cells that inherently possess a higher pluripotent capacity. This phenomenon should be further investigated to better understand the underlying mechanisms.

Additionally, these findings demonstrate that it is possible to obtain high-quality RNA from both microdevices (MDs) and macroscale samples, enabling further molecular studies. The increased expression of certain genes could be attributed to the presence of resistant cells with pluripotent capacity or as an adaptive response to the treatment. In either case, further investigation is needed to determine the biological relevance and implications of this increased expression in relation to treatment resistance.

### 3.4. Tracking Individual Spheres—Setting Growth Rate

**Figure 4:**
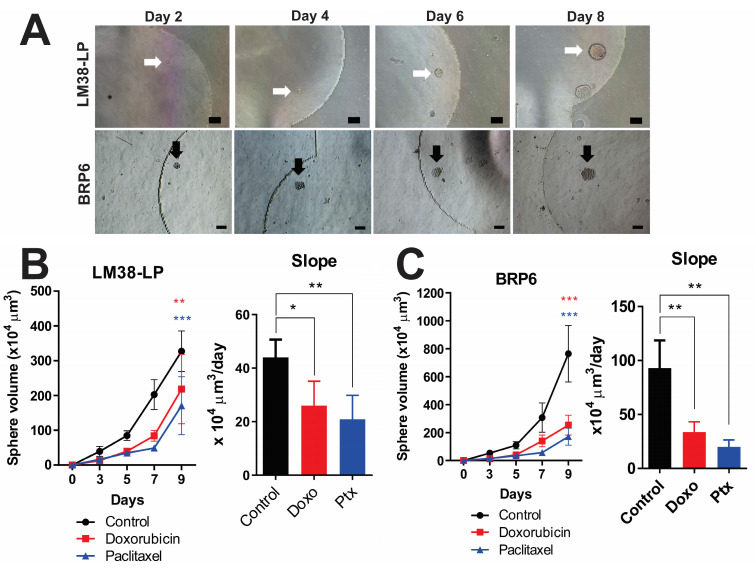
Monitoring the individual growth of tumor spheres under control and chemotherapeutic treatment conditions in MDs. (**A**) Representative images of LM38-LP and BRP6 spheres growing at different time points. Arrows indicate the spheres selected for growth monitoring. Bars represent the mean ± standard deviation from at least 4 independent experiments. (**B**,**C**) The curves were analyzed using two-way ANOVA and one-way ANOVA for slope analysis, both with Dunnett’s multiple comparisons (* *p* < 0.05, ** *p* < 0.005, *** *p* < 0.00005). Scale bar: 100 µm.

One of the advantages of MD is the ability to track individual cells and monitor their growth over time. Images of cultured spheres were captured every two days, enabling the measurement of their diameters, the calculation of sphere volumes, and the generation of growth curves. Figure 4A illustrates the time-course monitoring of a sphere derived from murine LM38-LP and human BRP6 cell lines under control conditions. This approach allows for the determination of growth rates for both control and treated spheres.

A notable reduction in both size and growth rate was observed in spheres treated with Doxo or Ptx after 8 days of culture, compared to their respective controls (Figure 4B,C). Specifically, the growth slope of treated spheres was lower than that of control spheres (Figure 4A,B). These findings are particularly significant, as this technique can be used to assess cell heterogeneity and the potential of cells to form tumors.

### 3.5. Sphere-Forming Ability of Murine and Canine Tumors Within the MD

So far, we have evaluated the use of MD as a culture platform for spheres derived from murine and human cancer cell lines. However, tumors are not composed solely of cancer cells; they also contain a microenvironment that can influence tumor growth and harbor varying amounts of CSC compared to pure cell lines.

To further investigate this complexity, cells derived from LM38-LP tumors growing in the breast of BALB/c mice were collected. The tumors were mechanically disaggregated, and cells were counted and seeded onto MD, enabling comparison with the growth of the pure cell line. Table 4 presents the number of spheres and their respective diameters for both the LM38-LP cell line and the primary tumor culture.

Our findings indicate that tumors generate fewer and smaller spheres compared to the pure cell line, suggesting a lower abundance of CSCs in the tumor microenvironment relative to pure cancer cell lines. However, treatment with Ptx significantly reduced the number and size of spheres in both the cell line and the tumor. These results suggest that MD is a valuable tool for assessing the therapeutic response of tumor-derived CSCs.

**Figure 5:**
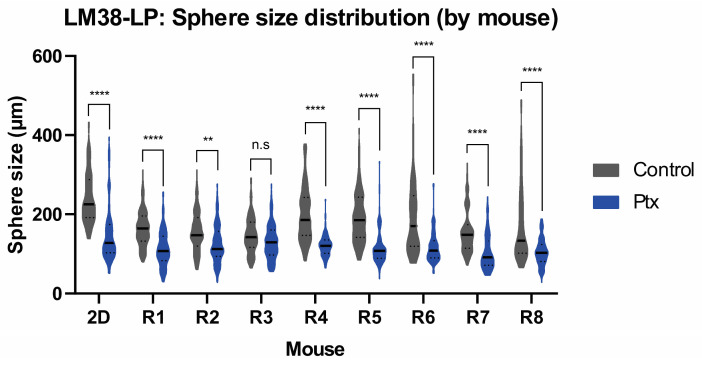
Growth of tumor spheres derived from primary LM38-LP tumor cultures in MDs. Sphere size distribution for each tumor (N = 8). Sphere sizes, based on average diameters per mouse, were analyzed using Student’s *t*-tests (** *p* < 0.005, **** *p* < 0.00005, n.s: not significant).

Figure 5 displays the size of spheres from eight individual tumors (R1 to R8), compared to the size of the pure cell line (2D) under control conditions or with Ptx. Treatment with Ptx resulted in a significant decrease in sphere size in seven out of eight tumors, demonstrating the reproducibility of the results. These findings support the validity of using MDs as a platform for isolating spheres and directly testing drugs on tumor samples. Consequently, this approach provides a more accurate representation of the culture of patient-derived tumors.

**Figure 6:**
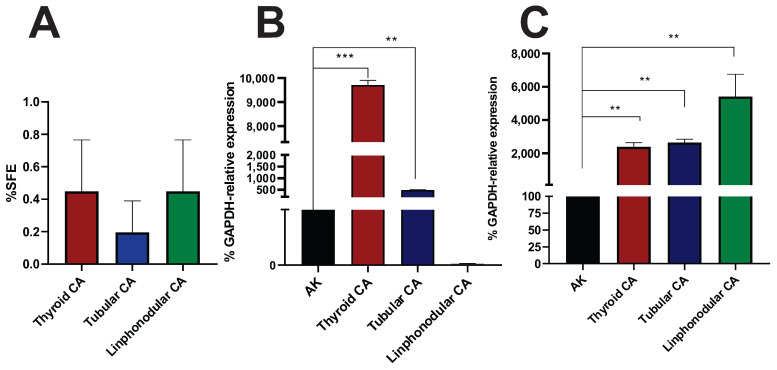
(**A**) Sphere formation efficiency (%SFE) for thyroid, tubular, and lymphonodular carcinoma and expression of pluripotency marker genes by qPCR in spheres cultured in MDs. (**B**) Relative mRNA expression of the Sox2 marker in spheres from different tumor types. (**C**) Relative mRNA expression of the Nanog marker in spheres from different tumor types. Bars represent the mean ± standard deviation from at least 3 independent experiments. Marker expression was compared using multiple *t*-tests per group (** *p* < 0.005, *** *p* < 0.0005).

To better approximate patient samples, we conducted studies on canine tumors. Three tumor samples, solid thyroid carcinoma, simple tubular carcinoma, and reactive lymph node, were processed through mechanical disaggregation, resulting in sphere growth within MD. As shown in Figure 6, it was possible to quantify the spheres and assess the SFE (Figure 6A). In all tumor types, an SFE of less than 1% was observed.

Additionally, the expression of pluripotency marker genes was measured, confirming the presence of a stem cell population. These results suggest that MD is a valuable tool for evaluating CSCs from veterinary tumor samples.

## 4. Discussion

For many years, tumors have been treated with cytostatic compounds derived from clinical trials. Patients were treated based on collective responses from these trials, yet not all responded satisfactorily. With the advent of molecular biology and knowledge of the human genome, significant progress has been made in understanding genomic alterations, particularly those associated with carcinogenesis and potential therapeutic targets [17,18]. Personalized medicine has gained significant relevance. It is now evident that even when tumors share certain genetic alterations, specific variations exist in each tumor and patient. Consequently, precision oncology requires tools capable of identifying which patients are more likely to benefit from specific treatments.

In this context, cancer stem cells (CSCs) have emerged as key players in therapy resistance and tumor recurrence. CSC represent a minority subpopulation within tumors, characterized by self-renewal and the ability to differentiate into various tumor cell types [19,20,21,22,23]. Targeting these cells is therefore crucial for achieving durable therapeutic responses and reducing relapse rates.

Methodologies using tumor samples to assess oncological responses hold promise for more effective and personalized treatments [24]. However, limited sample size often hinders the feasibility of multiple simultaneous assays. To overcome this limitation, we developed a CSC-based functional assay using a novel MD, which requires minimal sample and reagent volumes. The MD is equipped with six independent channels, each containing five cisterns, enabling the simultaneous testing of six experimental conditions with sufficient statistical power. The platform is compatible with immunofluorescence and RNA extraction, making it suitable for both morphological and molecular characterization.

Results obtained using this device show strong concordance with those from conventional macro-scale methodologies, underscoring its potential for translational applications. Importantly, mechanical and chemical stresses induced by microfluidic platforms can affect cell viability, membrane integrity, and the expression of stemness-associated genes. To mitigate these issues, we employed a manual seeding approach using an automatic micropipette, carefully controlling flow speed and angle to minimize turbulence as previously described [13,14]. This strategy resulted in a homogeneous distribution of spheres across all wells, demonstrating the consistency of our approach.

Additionally, to reduce stress during RNA extraction, we performed direct lysis within the MD. This in situ lysis protocol minimizes mechanical handling and helps preserve transcriptomic integrity. Collectively, these design and procedural considerations enhance the robustness, reproducibility, and accessibility of our platform, positioning it as a practical and low-cost tool for CSC investigation in low-resource laboratory settings.

In this study, we demonstrated that both murine and human breast cancer cell lines (LM38-LP and BRP6) successfully formed spheres in the MD and that treatment with doxorubicin or paclitaxel significantly reduced sphere size and growth rate, indicating effective targeting of proliferative cells. These observations underscore that conventional chemotherapy may fail to eradicate tumors due the persistence of resistant subpopulations, such as CSCs [25,26]. Interestingly, chemotherapy treatment was associated with an increase in pluripotent gene expression (Oct4, Nanog, Sox2, and CD44), assessed by both immunofluorescence and qPCR. This phenomenon raises two plausible, non-exclusive biological interpretations: (i) the selective survival and enrichment of pre-existing CSCs that are intrinsically resistant to treatment and (ii) the induction of stem-like traits in previously non-stem cancer cells as an adaptive response to stress or injury. Similar adaptive reprogramming has been described in glioblastoma [27], and small-cell lung cancer [28], where chemotherapy promotes cellular plasticity and dedifferentiation. Future lineage tracing and single-cell RNA-seq studies could help delineate these mechanisms in our model.

Furthermore, we validated the MD’s ability to support primary tumor cultures. CSC-enriched spheres were generated from orthotopic LM38-LP tumors in BALB/c mice. As expected, these spheres were smaller and less proliferative than those from cell lines, yet still responded to paclitaxel, reinforcing the clinical relevance of primary material. Importantly, we extended this approach to veterinary oncology, analyzing three canine tumors (thyroid carcinoma, tubular carcinoma, and lymphocytic carcinoma). All samples successfully formed spheres within the MD and showed elevated Nanog expression. This is consistent with reports that CSC-like populations can be isolated from canine tumors, including mammary and prostate carcinomas [29,30,31,32].

The ability to culture spheres from veterinary tumors not only validates the MD’s cross-species applicability but also opens avenues for comparative oncology—a field that integrates human and veterinary cancer research to enhance translational outcomes. The use of spontaneous canine tumors, which often share histological, molecular, and therapeutic features with human cancers, may provide a more representative model for preclinical studies than xenografts [33,34].

Despite promising results, several limitations must be acknowledged. First, the small sample size, particularly in veterinary cases, limits the generalizability of the findings. Second, the sphere formation assay, while widely used for CSC enrichment, remains an artificial in vitro model that does not fully replicate the in vivo tumor microenvironment as described in [35]. However, when primary cultures are performed within the MDs, the tumor microenvironment is better represented, constituting an important added value. Third, CSC marker expression (e.g., Oct4 and Nanog) can be influenced by culture conditions and is not always exclusive to stem-like populations, which may lead to interpretive biases. We acknowledge the limited number of veterinary tumor samples analyzed in this study, which prevents us from generalizing that all tumor types will form spheres under our culture conditions. For instance, while canine mastocytomas were capable of growing in 2D monolayer cultures, they failed to generate spheres. This observation raises two important considerations: first, not all tumor types may harbor progenitor or CSCs with sphere-forming potential; second, and of particular relevance for the translational applicability of the MD platform, is the need for a broader clinical evaluation. Such studies would help determine which tumor types are amenable to growth and CSC enrichment under these conditions and assess how well the in vitro responses correlate with clinical outcomes.

Taking these limitations into account, the present study should be viewed as a proof of concept for the use of microdevices in the isolation, characterization, and functional assessment of CSCs from minimal tumor material, with potential application in personalized oncology and veterinary cancer research.

To further validate the platform, future studies should include the following: (a) expansion to a larger and more diverse cohort of human and veterinary tumors, (b) drug screening with panels of chemotherapeutic agents and targeted therapies, (c) integration with patient-derived organoids or xenografts for longitudinal correlation with clinical outcomes, (d) and high-throughput single-cell profiling to unravel the heterogeneity of treatment responses.

This approach can enhance the understanding of cancer treatment responses and has the potential to improve patient-specific therapies. In the future, scaling up this methodology will enable the performance of mixed co-cultures of tumor cells with immune system cells or fibroblasts, allowing for the analysis of immunomodulatory therapies and other treatment strategies.

In summary, this work represents a proof-of-concept for using microfluidic devices to isolate and evaluate CSCs from small tumor samples. The MD enables rapid and multiplexed assessment of treatment response, supports the culture of primary tumor material, and may serve as a powerful tool for functional diagnostics in precision oncology.

## 5. Conclusions

Taken together, these results highlight the relevance of CSC analysis as a central component in both precision oncology and the study of cancer stem cell biology. Furthermore, our findings demonstrate the versatility of the microdevice platform, which integrates several key features for this type of research, including minimal sample and reagent requirements, and a culture environment well-suited for sphere formation from murine and human tumor cell lines. Notably, we achieved CSC-enriched cultures not only from established models but also from primary murine tumors and canine patient samples, underscoring the potential of this system as a predictive platform for evaluating therapeutic response in both experimental and clinical oncology settings.

## Figures and Tables

**Table 1:** Oligonucleotide murine and human primers.

*Gene*	*Forward Primer (5′→3′)*	*Reverse Primer (5′→3′)*	*Amplicon Size (bp)*
**mOct4**	TCAGGTTGGACTGGGCCTAGT	GGAGGTTCCCTCTGAGTTGCTT	100
**hOct4**	GTGGAGGAAGCTGACAACAATG	AATTCTCCAGGTTGCCTCTCATC	121
**mSox2**	CCTCCGGGACATGATCAGCATGTA	GCAGTGTGCCGTTAATGGCCGTG	131
**hSox2**	GAGCTTTGCAGGAAGTTTGC	GCAAGAAGCCTCTCCTTGAA	190
**mNanog**	GAAATCCCTTCCCTCGCCATC	CTCAGTAGCAGACCCTTGTAAGC	161
**hNanog**	ACAACTGGCCGAAGAATAGCA	GGTTCCCAGTCGGGTTCAC	111
**mCD44**	AAAAAGCCATGCAGCAGCTC	TTGCCTCTTGGGTGGTGTTT	145
**hCD44**	CGGACACCATGGACAAGTTT	GAAAGCCTTGCAGAGGTCAG	176
**mGAPDH**	CAAAATGGTGAAGGTCGGTG	CAATGAAGGGGTCGTTGATG	113
**hGAPDH**	ACCCACTCCTCCACCTTTGA	CTGTTGCTGAGCCAAATTCGT	100
**cSox2**	AACCCCAGATGCACAACTC	CGGGGCCGGTATTTATAATC	162
**cNanog**	GAATAACCCGAATTGGAGCAG	AGCGATTCCTCTTCACAGTTG	141
**cGAPDH**	TGACACCCACTCTTCCACCTTC	CGGTTGCTGTAGCCAAATTCA	105

Oligonucleotide murine and human primers used for real-time PCR analysis; m: murine; h: human; c: canine.

**Table 2:** SFE and sphere size from LM38-LP and BRP6 cell lines.

	*%SFE*			*Sphere Size (µm)*		
	*LM38-LP (MW-12)*	*LM38-LP (MD)*	*BRP6* *(MD)*	*LM38-LP (MW-12)*	*LM38-LP* *(MD)*	*BRP6* *(MD)*
*Control*	28.3 ± 1.42	13.2 ± 1.2	10.5 ± 0.8	133.3 ± 75.1	192.7 ± 67.3	182.1 ± 77.4
*Doxo*	16.7 ± 0.7 (***)	8.1 ± 2.7 (**)	6.4 ± 1.83 (**)	98.9 ± 54.6 (**)	103.1 ± 32.7 (****)	133.7± 55.2 (***)
*Ptx*	21.2 ± 1.91 (**)	7.2 ± 2.7 (**)	4.2 ± 0.89 (***)	108.2 ± 55.2 (**)	118.3 ± 39.9 (***)	127.1 ± 49.3 (***)

SFE and sphere size from LM38-LP and BRP6 cell lines seeded in MDs and multiwell (MW12) culture plate and treated with doxorubicin or paclitaxel for 7 days. One-way block ANOVA from 3 independent experiments with four replications was made (**** *p* < 0.0001, *** *p* < 0.0005, ** *p* < 0.05).

**Table 3:** Pluripotency genetic markers expression.

			#cells/mL (×10^3^)	Total RNA (μg)	Oct4	Sox2	Nanog	CD44
LM38-LP	MD	Control	100	17.4 ± 1.9	100	100	100	100
		Doxo	100	4.6 ± 0.8	189.1 ± 54.1 (**)	210.7 ± 101.2 (***)	183.5 ± 63.4 (***)	350.9 ± 81.1 (***)
		Ptx	100	5.8 ± 2.1	161.9 ± 55.2 (**)	242.1 ± 81.5 (***)	177.9 ± 50.1 (**)	403.1 ± 7.8 (***)
	MW-12	Control	3	81.4 ± 17.2	100	100	100	100
		Doxo	3	53.7 ± 11.5	1308.1 ± 558.9 (***)	1087.1 ± 356.9 (***)	858.4 ± 342.9 (***)	486.3 ± 199.9 (*)
		Ptx	3	51.8 ± 19.8	852.6 ± 452.8 (***)	419.7 ± 183.30 (*)	771.6 ± 287.3 (***)	918.3 ± 263.6 (***)
BRP6	MD	Control	100	13.6 ± 1.5	100	100	100	100
		Doxo	100	6.4 ± 1.1	386.17 ± 48.3 (***)	556.3 ± 192.2 (**)	313.2 ± 144.2 (*)	351.5 ± 133.4 (**)
		Ptx	100	5.6 ± 0.9	410.84 ± 62.7 (***)	416.2 ± 130.8 (**)	346.2 ± 148.6 (*)	457.7 ± 94.4 (***)

Pluripotency genetic markers expression. Quantification of mRNA levels for Oct4, Sox2, Nanog, and CD44 in LM38-LP and BRP6 spheres was performed by qPCR, as described in the Materials and Methods section. Results are expressed relative to the untreated control group (set at 100%) for each gene. Statistical analysis was conducted using one-way ANOVA followed by Dunnett’s multiple comparisons test. Data represent the mean ± standard deviation from three independent experiments, each with four technical replicates. (*** *p* < 0.0005, ** *p* < 0.005, and * *p* < 0.05).

**Table 4:** Comparison of SFE and sphere size between primary tumor LM38-LP culture cell and pure LM38-LP cell line.

	*#spheres*		*Sphere Size (µm)*	
	*Cell Line*	*Tumor*	*Cell Line*	*Tumor*
*Control*	140 ± 9	89 ± 18	243.2 ± 68.03	174.3 ± 72.85
*Ptx*	78 ± 29 (***)	59 ± 20 (*)	152.3 ± 73.76 (***)	117 ± 42.90 (***)

Comparison of SFE and sphere size between primary tumor LM38-LP culture cell and pure LM38-LP cell line. The cultures of eight independent tumors were evaluated in duplicate and compared with the pure LM38-LP line. All experiments were analyzed using Student’s *t*-tests (* *p* < 0.05, *** *p* < 0.0005).

## Data Availability

Datasets generated and analyzed during the current study are available from the corresponding author upon reasonable request. Due to ethical and legal restrictions, raw data derived from patient and veterinary tumor samples are not publicly available to protect privacy and comply with institutional regulations.

## Abbreviations

The following abbreviations are used in this manuscript:

CSCs	Cancer Stem Cells
MD	Microdevice
CT	Chemotherapy
SFE	Sphere-Forming Efficiency
LOC	Lab-On-A-Chip
IF	Immunofluorescence
CM	Complete Medium
Doxo	Doxorubicin
Ptx	Paclitaxel
PDMS	Polydimethylsiloxane
SFM	Serum-Free Medium

## References

[1] Bray F., Laversanne M., Sung H., Ferlay J., Siegel R.L., Soerjomataram I., Jemal A. (2024). Global cancer statistics 2022: GLOBOCAN estimates of incidence and mortality worldwide for 36 cancers in 185 countries. CA A Cancer J. Clin..

[2] Zhou H.M., Zhang J.G., Zhang X., Li Q. (2021). Targeting cancer stem cells for reversing therapy resistance: Mechanism, signaling, and prospective agents. Signal Transduct. Target. Ther..

[3] Vasan N., Baselga J., Hyman D.M. (2019). A view on drug resistance in cancer. Nature.

[4] Holohan C., Van Schaeybroeck S., Longley D.B., Johnston P.G. (2013). Cancer drug resistance: An evolving paradigm. Nat. Rev. Cancer.

[5] Liu X., Li G., Liu Q., Guo Z., Li B., Zhang Y., Li X., Wang J. (2023). Cancer stem cells and their niche in cancer progression and therapy. Cancer Cell Int..

[6] Yang L., Shi P., Zhao G., Xu J., Peng W., Zhang J., Zhang G., Wang X., Dong Z., Chen F. (2020). Targeting cancer stem cell pathways for cancer therapy. Signal Transduct. Target. Ther..

[7] Vidal S.J., Rodriguez-Bravo V., Galsky M., Cordon-Cardo C., Domingo-Domenech J. (2014). Targeting cancer stem cells to suppress acquired chemotherapy resistance. Oncogene.

[8] Zhu F., Qian W., Zhang H., Liang Y., Wu M., Zhang Y., Zhang X., Gao Q., Li Y. (2017). SOX2 Is a Marker for Stem-like Tumor Cells in Bladder Cancer. Stem Cell Rep..

[9] Chaudhary A., Raza S.S., Haque R. (2023). Transcriptional factors targeting in cancer stem cells for tumor modulation. Semin. Cancer Biol..

[10] Tsujii M. (2014). [Cancer therapy targeting cancer stem cell]. Nihon Rinsho..

[11] Lytle N.K., Barber A.G., Reya T. (2018). Stem cell fate in cancer growth, progression and therapy resistance. Nat. Rev. Cancer.

[12] Magee J.A., Piskounova E., Morrison S.J. (2012). Cancer Stem Cells: Impact, Heterogeneity, and Uncertainty. Cancer Cell.

[13] Belgorosky D., Fernández-Cabada T., Peñaherrera-Pazmiño A.B., Langle Y., Booth R., Bhansali S., Pérez M.S., Eiján A.M., Lerner B. (2018). Analysis of tumoral spheres growing in a multichamber microfluidic device. J. Cell. Physiol..

[14] Agüero E.I., Belgorosky D., García-Silva J.I., Booth R., Lerner B., Pérez M.S., Eiján A.M. (2023). Correction to: Microdevices for cancer stem cell culture as a predictive chemotherapeutic response platform. J. Mol. Med..

[15] Bumaschny V., Urtreger A., Diament M., Krasnapolski M., Fiszman G., Klein S., Joffé E.B.d.K. (2004). Malignant myoepithelial cells are associated with the differentiated papillary structure and metastatic ability of a syngeneic murine mammary adenocarcinoma model. Breast Cancer Res..

[16] Reyes-Moreno C., Girouard J., Marino L., Belgorosky D., Langle Y.V., Hamelin-Morrissete J., Agüero E.I., Malagrino H., Eiján A.M. (2020). Relevance of iNOS expression in tumor growth and maintenance of cancer stem cells in a bladder cancer model. J. Mol. Med..

[17] Herrera M., Berral-González A., López-Cade I., Galindo-Pumariño C., Bueno-Fortes S., Martín-Merino M., Carrato A., Ocaña A., De La Pinta C., López-Alfonso A. (2021). Cancer-associated fibroblast-derived gene signatures determine prognosis in colon cancer patients. Mol. Cancer.

[18] Dankner M., Rose A.A.N., Rajkumar S., Siegel P.M., Watson I.R. (2018). Classifying BRAF alterations in cancer: New rational therapeutic strategies for actionable mutations. Oncogene.

[19] Chu X., Tian W., Ning J., Xiao G., Zhou Y., Wang Z., Zhai Z., Tanzhu G., Yang J., Zhou R. (2024). Cancer stem cells: Advances in knowledge and implications for cancer therapy. Signal Transduction and Targeted Therapy.

[20] Tannishtha R., Morrison S.J., Clarke M.F., Weissman I.L. (2001). Stem cells, cancer, and cancer stem cells. Nature.

[21] Clevers H. (2011). The cancer stem cell: Premises, promises and challenges. Nat. Med..

[22] El-Tanani M., Rabbani S.A., Satyam S.M., Rangraze I.R., Wali A.F., El-Tanani Y., Aljabali A.A.A. (2025). Deciphering the Role of Cancer Stem Cells: Drivers of Tumor Evolution, Therapeutic Resistance, and Precision Medicine Strategies. Cancers.

[23] Zhang S., Yang R., Ouyang Y., Shen Y., Hu L., Xu C. (2024). Cancer stem cells: A target for overcoming therapeutic resistance and relapse. Cancer Biol. Med..

[24] Friedman A.A., Letai A., Fisher D.E., Flaherty K.T. (2016). Precision medicine for cancer with next-generation functional diagnostics. Nat. Rev. Cancer.

[25] Shibata M., Hoque M.O. (2019). Targeting cancer stem cells: A strategy for effective eradication of cancer. Cancers.

[26] Zhang C., Semenza G.L., Zhang H., Shimoda L.A., Lu H., Chen I., Park Y., Tran L. (2017). Chemotherapy-Induced Ca2+ Release Stimulates Breast Cancer Stem Cell Enrichment. Cell Rep..

[27] Guerra-Rebollo M., Garrido C., Sánchez-Cid L., Soler-Botija C., Meca-Cortés O., Rubio N., Blanco J. (2019). Targeting of replicating CD133 and OCT4/SOX2 expressing glioma stem cells selects a cell population that reinitiates tumors upon release of therapeutic pressure. Sci. Rep..

[28] Bang J.S., Choi N.Y., Lee M., Ko K., Park Y.S., Ko K. (2019). Reprogramming of cancer cells into induced pluripotent stem cells questioned. Int. J. Stem Cells.

[29] Navarro-Marchal S.A., GrinÌ án-Lisan C., Entrena J.M., Ruiz-Alcalá G., Tristán-Manzano M., Martin F., Pérez-Victoria I., Peula-Garciá J.M., Marchal J.A. (2021). Anti-CD44-Conjugated Olive Oil Liquid Nanocapsules for Targeting Pancreatic Cancer Stem Cells. Biomacromolecules.

[30] Bocci F., Levine H., Onuchic J.N., Jolly M.K. (2019). Deciphering the Dynamics of Epithelial-Mesenchymal Transition and Cancer Stem Cells in Tumor Progression. Curr. Stem Cell Rep..

[31] Król M., Rybicka A. (2016). Identification and characterization of cancer stem cells in canine mammary tumors. Acta Veter- Scand..

[32] Pang L.Y., Cervantes-Arias A., Else R.W., Argyle D.J. (2011). Canine mammary cancer stem cells are radio- and chemo- resistant and exhibit an epithelial-mesenchymal transition phenotype. Cancers.

[33] Michishita M., Akiyoshi R., Yoshimura H., Katsumoto T., Ichikawa H., Ohkusu-Tsukada K., Nakagawa T., Sasaki N., Takahashi K. (2011). Characterization of spheres derived from canine mammary gland adenocarcinoma cell lines. Res. Veter.-Sci..

[34] Kishimoto T.E., Yashima S., Nakahira R., Onozawa E., Azakami D., Ujike M., Ochiai K., Ishiwata T., Takahashi K., Michishita M. (2017). Identification of tumor-initiating cells derived from two canine rhabdomyosarcoma cell lines. J. Veter.-Med. Sci..

[35] Zhao H., Yan C., Hu Y., Mu L., Huang K., Li Q., Li X., Tao D., Qin J. (2019). Sphere-forming assay vs. Organoid culture: Determining long-term stemness and the chemoresistant capacity of primary colorectal cancer cells. Int. J. Oncol..

